# Individual Differences in Temporal Selective Attention as Reflected in Pupil Dilation

**DOI:** 10.1371/journal.pone.0145056

**Published:** 2015-12-14

**Authors:** Charlotte Willems, Johannes Herdzin, Sander Martens

**Affiliations:** 1 Department of Neuroscience, University Medical Center Groningen, Groningen, the Netherlands; 2 Neuroimaging Center, University of Groningen, Groningen, the Netherlands; University of Verona, ITALY

## Abstract

**Background:**

Attention is restricted for the second of two targets when it is presented within 200–500 ms of the first target. This attentional blink (AB) phenomenon allows one to study the dynamics of temporal selective attention by varying the interval between the two targets (T1 and T2). Whereas the AB has long been considered as a robust and universal cognitive limitation, several studies have demonstrated that AB task performance greatly differs between individuals, with some individuals showing no AB whatsoever.

**Methodology/Principal Findings:**

Here, we studied these individual differences in AB task performance in relation to differences in attentional timing. Furthermore, we investigated whether AB magnitude is predictive for the amount of attention allocated to T1. For both these purposes pupil dilation was measured, and analyzed with our recently developed deconvolution method. We found that the dynamics of temporal attention in small versus large blinkers differ in a number of ways. Individuals with a relatively small AB magnitude seem better able to preserve temporal order information. In addition, they are quicker to allocate attention to both T1 and T2 than large blinkers. Although a popular explanation of the AB is that it is caused by an unnecessary overinvestment of attention allocated to T1, a more complex picture emerged from our data, suggesting that this may depend on whether one is a small or a large blinker.

**Conclusion:**

The use of pupil dilation deconvolution seems to be a powerful approach to study the temporal dynamics of attention, bringing us a step closer to understanding the elusive nature of the AB. We conclude that the timing of attention to targets may be more important than the amount of allocated attention in accounting for individual differences.

## Introduction

Although human beings can extract the gist of a visual scene within a fraction of a second, they can be relatively slow to select sequentially presented relevant information from a rapid stream of irrelevant information. That is, attention to relevant information is restricted for the second of two targets when it is presented within 200–500 ms of the first target. This phenomenon, known as the attentional blink (AB; [1]), allows one to study the dynamics of temporal selective attention by systematically varying the interval between the two targets (T1 and T2). Recent evidence suggests that the AB is due to the default use of an adverse attentional strategy [2–6], but the precise nature of this problem to control attention remains unclear.

To shed light on this underlying mechanism of the AB, one can study the origin of individual differences in AB task performance; Although the AB was initially considered to reflect a universal, fundamental attentional restriction, large differences in individual AB task performance have been revealed over the past years [7–13]. In these studies, individual AB task performance has been investigated in relation to a variety of factors, e.g., other cognitive tasks [13–15], personality characteristics [16, 17], and lifestyle [18, 19], either within a large group of participants [10, 20–22], or by contrasting performance of two extreme groups, i.e., blinkers vs. non-blinkers; [9, 23].

### Timing attention

One difference underlying individual AB task performance may be the timing of attention allocated to the targets as presented in the rapid serial visual presentation (RSVP) stream. Although processing speed per se has not been found to be a predictive factor for individual AB magnitude [24, 25], studies have revealed individual differences in attentional timing in the AB paradigm; As indicated by the event related potential component P3 in trials where both targets were identified correctly, i.e., no-blink trials, it was revealed that non-blinkers are earlier in updating the content of working memory (WM) than blinkers, irrespective of lag or target [9, 16]. In addition, it was found that individuals with a higher rate of WM updating showed a larger AB magnitude [26]. Furthermore, we have shown that the size of the AB was predictive for the precision of target selection [21]. That is, in an AB task where the stream of stimuli contained letters only, and targets and distractors had to be distinguished based on color, small blinkers selected items that were close to the actual target whereas the selection pattern was more diffused for large blinkers. Here, we also revealed that a smaller AB magnitude is related to better preservation of temporal order information, as reflected in fewer reversed order reports of T1 and T2 [21].

To reveal the temporal dynamics of attentional allocation in the AB paradigm, we have recently focused on pupil dilation as a measure of attentional timing and cognitive workload [27]. However, because the pupil response takes ~1 sec, responses to stimuli in a fast-paced task like the AB task are overlapping, and any meaningful differences remain concealed. Therefore, the pupil dilation deconvolution method was developed, which allows one to isolate and track the temporal dynamics of target-related attentional allocation [27]. Because the total pupil response to the RSVP stream is assumed to be the sum of all separate responses to the stimuli [28], the signal can be deconvolved to single pulses related to attention allocated to the presented stimuli [27]. Earlier, using the pupil dilation deconvolution method to track training-induced changes in attentional allocation, we already found that better task performance in the AB paradigm was related to earlier attentional allocation to T2, but there was no such evidence for T1 [29].

In the current study, the first aim is to reveal the time course of attentional allocation in the AB paradigm in relation to individual AB magnitude. By analyzing reversed order reports of T1 and T2, we will test whether we can replicate our earlier findings that better preservation of temporal order information is related to a smaller AB magnitude [21]. Moreover, by measuring pupil dilation, we will focus on differences in attentional timing to the targets in relation to individual AB task performance. Because small blinkers appear to be earlier in WM updating [9, 16], and more efficient in target selection [21, 24], we hypothesize that earlier allocation of attention will be related to a smaller AB magnitude. In the deconvolved pupil data, this would result in earlier peaks of the pulses reflecting attentional allocation to the targets. Based on our previous pupil dilation study [29], we expect that this relation between earlier attentional allocation and better AB task performance will be most pronounced for T2.

### Overinvestment of attention

Next to timing, the strength of attentional investment can also be studied by measuring pupil dilation. In previous studies, it has been argued that one aspect of the detrimental strategy that presumably causes the AB is attentional overinvestment to T1 [4, 30, 31]. That is, if cognitive control is deployed too stringent towards target selection, an overly strong focus on selecting the first target is likely to come at a cost for selecting the second target during the AB period. This theory was supported by earlier pupil dilation studies, where the attentional response to T1 was found higher in blink trials than in no-blink trials [27, 29]. Further evidence was obtained in an EEG study, reporting a higher P3 amplitude in blink trials than in no-blink trials [8]. However, these studies found no relation between individual AB task performance and attentional investment to T1 [8, 10, 29]. In contrast, though, both an fMRI and a MEG study showed that higher activation related to T1 encoding was linked to lower individual T2 accuracy [30, 32].

To resolve these somewhat mixed findings, the second goal of this study is to examine the amount of attention allocated to the targets, as indicated by the deconvolved pupil signals. Hereby, we aim to reveal whether any attentional overinvestment to T1 is related to individual AB task performance. It is expected that attentional overinvestment to T1 will result in higher T1 amplitudes of the deconvolved pulses in blink trials compared to no-blink trials. In addition, if any attentional overinvestment is related to individual AB magnitude, a larger AB magnitude is expected to be related to a larger attentional response to T1.

In summary, the aim of the current study is twofold: First, we aim to reveal the time course of individual attentional deployment in the AB paradigm. Second, we will investigate whether AB magnitude is predictive for the amount of attention allocated to T1. For both these purposes pupil dilation will be measured.

## Methods

### Participants

The study was approved by the Psychology Ethical Committee of the University of Groningen, and participants signed a written informed consent form prior to the experiment. In total, 100 students performed the experiment for which they received course credits in return. The experiment was performed together with an experiment on temporal integration that will be reported elsewhere. The order of these two experiments was counterbalanced, and together, these experiments were completed in ~90 minutes. The duration of the current experiment was ~30 minutes. After initial data screening, ten participants were excluded, because either T1 accuracy was < 50%, or data logging went wrong. Regarding the analyses of the pupil data, another nine participants were excluded, because the pupil data contained too many artifacts—more than one third of the trials had to be discarded—or pupil measurement did not succeed. This left 90 participants (55 women; mean age = 20.45, ranging 18–29) for the behavioral analyses, and 81 participants (52 women; mean age = 20.48, ranging 18–29 years) for the pupil analyses.

### Apparatus and stimuli

The experiment was generated and recorded by E-prime 2.0 software, and presented in the middle of a 19-inch CRT monitor with a 100 Hz refresh rate. Target stimuli were uppercase consonants, excluding “Q”, “X”, and “Y”, whereas distractor stimuli were digits, excluding “0” and “1”. Stimuli were presented in black, 18-point Courier New on a white background. Pupil size was measured using the EyeLink 1000 eye-tracker (www.sr-research.com) at a sampling rate of 250 Hz. Participants kept their head in a chin-rest during the experiment and viewing distance was ~50 cm.

### Procedure

The experiment contained a practice block of 20 trials, and a test block of 196 trials. Each trial started with a fixation cross of 1000 ms, followed by an RSVP of 32 stimuli (~10 Hz). In 168 of the 196 trials, the RSVP stream contained two target letters, i.e., dual-target trials, whereas in 28 trials, only one letter was presented, i.e., single-target trials. Taking these rates into account, trials were presented randomly with the additional constraint for dual-target trials that each lag was presented equally often. T1 was always the sixth item in the stream, which was chosen to allow for a consistent response to the first target, reducing unnecessary variability in behavioral performance and pupil dilation responses. In dual-target trials, T2 was presented as the first, third, or eighth item after T1, lag 1, 3, and 8, respectively. Stimuli were chosen randomly under the prerequisite that a target letter was never repeated within one trial, and that successive distractor digits were never the same. The RSVP was followed by either a 100-ms dot or comma, which had to be identified in addition to the target letters. This comma/dot task was included to encourage participants to remain fixated to the center of the screen throughout stimulus presentation, allowing optimal measurement of the pupil response to both targets. After each RSVP stream, participants where prompted to enter the target letters in the order they had seen them, using the corresponding keys on the keyboard, and to enter whether they had seen a comma or a dot. When a single or no letter was seen, participants could indicate this by pressing the space bar.

### Pre-processing pupil data

The pupil data were down-sampled to 50 Hz and time-locked to the onset of T1. The average pupil size in the 200 ms preceding the stream was used as baseline, and data were normalized by subtracting the baseline from the measured size and by dividing this value by the baseline. With the pupil dilation deconvolution method [27], per combination of participant and condition, 80 pulses were modeled, starting 260 ms before stream onset. The set of pulse strengths *i* was convolved with the Erlang gamma function $h\;=\;s*t(n)*e{\;}^{\left(\frac{-n*t}{\;t_{max}}\right)}$, where *s* is a scaling factor, *n* is the number of layers, and *t*_max_ is the position of the maximum response. These parameters were set to *n* = 10.1, t_max_ = 930 and *s* = 1/1027 [27]. The pulse strengths were obtained by optimizing the fit between the estimated signal *x* = *l* * *b* + *i* * *h* and the measured pupil dilation signal, where *l* is the position of each pulse in vector *i* and *b* controls for linear drifts in the data. As in [29], we used an inter-pulse interval of 50 ms, and the Levenberg-Marquardt algorithm for optimizing the strengths of the attentional pulses. Segments containing eye blinks were semi-automatically corrected using linear interpolation or discarded.

The latency of the pulse associated with T1 was determined by calculating the first local peak within a time window ranging -100 to 500 ms relative to the onset of T1. For T2, the latency was determined by calculating the local peak within a time frame ranging 500 to 1100 ms for lag 3, and 1000 to 1600 ms for lag 8, relative to the onset of T1. The average strength of the pulse preceding and the pulse following the local peak, and the local peak itself were used as a measure of amplitude.

### Statistical methods

Statistical analyses were performed using R (version 2.14.2; [33]). With the *lmerTest* package [34], behavioral data were analyzed with generalized linear mixed models (GLMM) and pupil dilation data with either linear mixed models (LMM) or, in case of no repeated measures, linear models (LM). To account for repeated measures, “participants” was entered in all mixed models as random intercept, and in case of overdispersion, an observation-level random intercept was added to the model. Continuous factors were standardized by subtracting the column means, and dividing these centered values by their standard deviations. Unless mentioned otherwise, fixed factors and interaction terms were included based on model comparisons using analyses of variance (ANOVA). To avoid collinearity between fixed factors, we tested different measures of AB task performance in different models. Covariance structures were modeled using the *nlme* package [35] and compared using the Akaike information criterion [36]. This comparison revealed that in all cases the structure with the assumption that there are no within-group correlations fitted best.

## Results

### Behavioral

**Fig 1:**
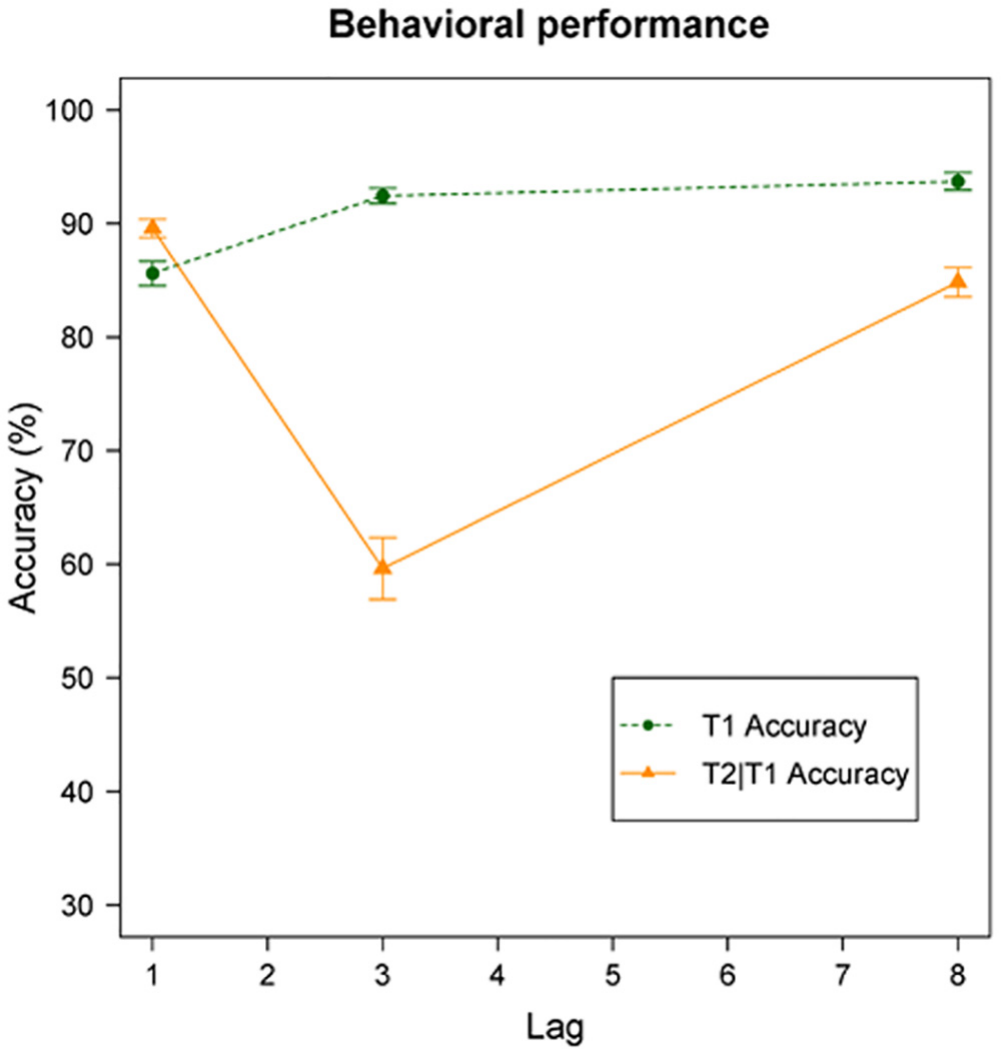
Mean accuracy AB task. Mean T1 accuracy and mean T2 accuracy assumed that T1 was identified correctly (T2|T1). The error bars reflect the standard errors of the mean.

**Fig 2:**
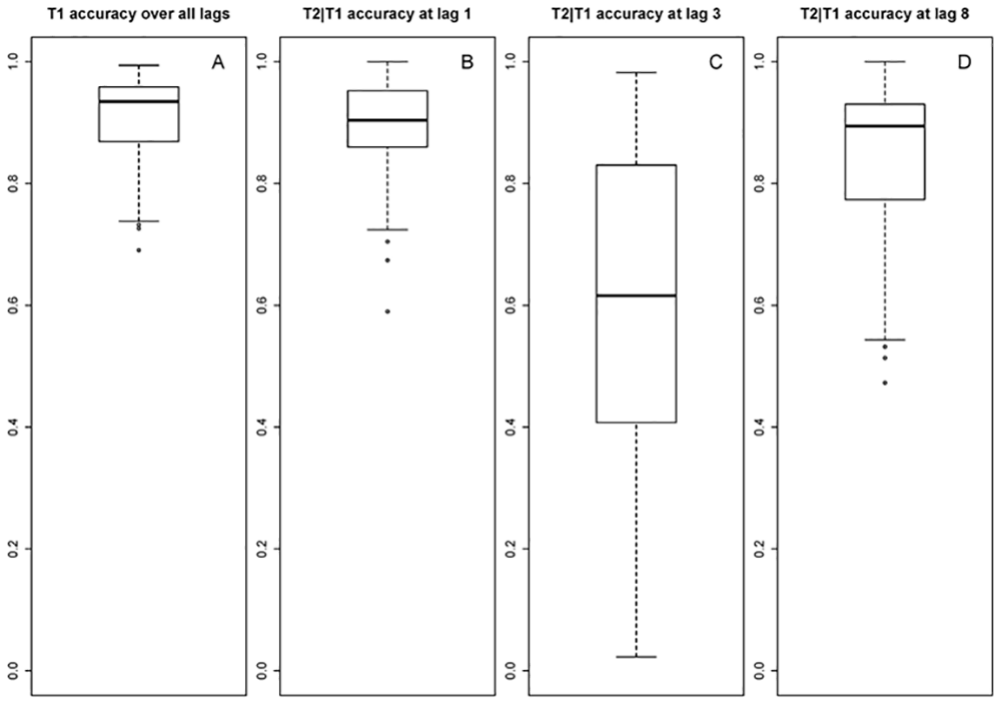
Individual performances AB task. Boxplots depicting the distribution of individual differences in the AB task A) for mean T1 accuracy over all lags (lag 1, 3, and 8), and for mean T2|T1 accuracy at B) lag 1, C) lag 3, and D) lag 8.

The behavioral results for T1, and T2 given correct report of T1 (T2|T1) are graphed in Fig 1. Mean T1 accuracy was 85.62% at lag 1, 92.44% at lag 3, and 93.71% at lag 8, and individual mean T1 accuracy over these lags ranged from 69.05% to 99.40%. The distribution of individual differences for mean T1 accuracy is displayed in the boxplot in Fig 2A. T1 accuracy was found to differ significantly between lag 1 and 3, *β* = .77, *SE* = .07, *z* = 11.18, *p* < .001; lag 1 and 8, *β* = .98, *SE* = .07, *z* = 13.44, *p* < .001; and lag 3 and 8, *β* = .21, *SE* = .08, *z* = 2.57, *p* = .010. Single-target accuracy was 94.25%.

Mean T2|T1 accuracy was 90.03% at lag 1, 60.25% at lag 3, and 85.43% at lag 8. Individual T2|T1 accuracy at lag 3, thus, within the AB period, ranged from 2.27% to 98.21%. The distribution of mean individual T2|T1 accuracy per lag is graphed in Fig 2B–2D. T2|T1 accuracy differed significantly between lag 1 and 3, *β* = -1.97, *SE* = .06, *z* = -31.40, *p* < .001; lag 1 and 8, *β* = -.44, *SE* = .07, *z* = -6.42, *p* < .001; and lag 3 and 8, *β* = 1.53, *SE* = .05, *z* = 27.97, *p* < .001. Trials where T1 and T2 were identified correctly, but reported in reversed order, were also counted as correct.

To determine AB magnitude, we calculated AB magnitude relative to mean T1 performance (meanT1 –T2|T1_lag3_ / meanT1), as well as AB magnitude relative to T2|T1 at lag 8 (T2|T1_lag8_ –T2|T1_lag3_ / T2|T1_lag8_). AB magnitude relative to mean T1 ranged from -.002 to .97 (*mean* = .35, *SE* = .002), whereas AB magnitude relative to lag-8 T2|T1 accuracy ranged from -.12 to .97 (*mean* = .31, *SE* = .002). Because these two measures correlated highly (Pearson’s *r* = .96, *p* < .001), we will only report the effects of AB magnitude relative to lag-8 T2|T1 accuracy [37].

**Fig 3:**
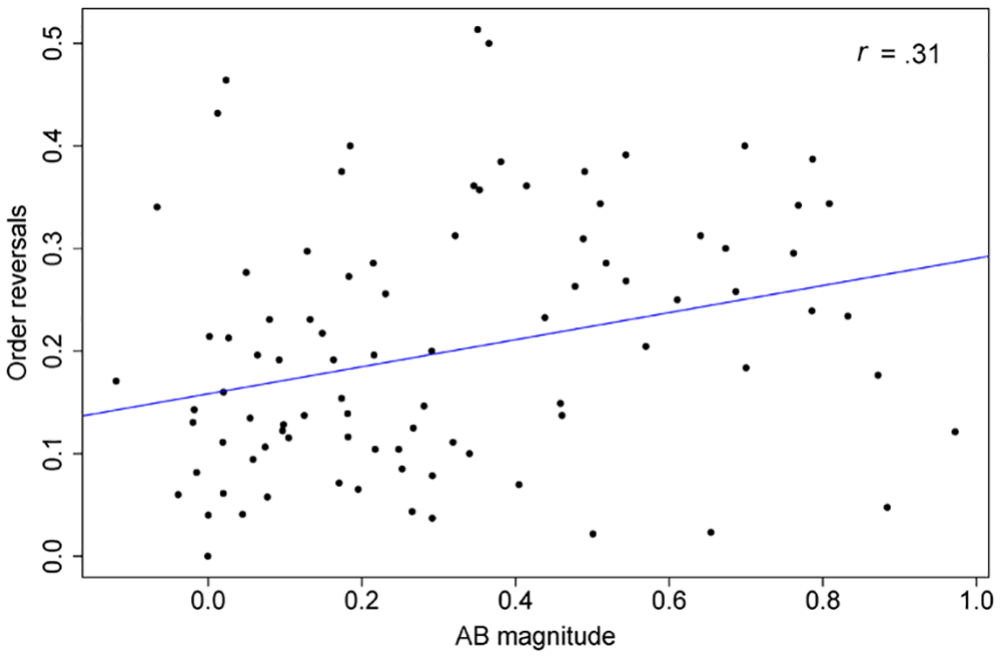
AB magnitude vs reversed order reports. Scatterplot depicting the relation between AB magnitude and the number of reversed order reports of T1 and T2 per individual in lag-1 no-blink trials. The blue line depicts a simple linear regression line between AB magnitude and order reversals. The correlation presented is a Spearman Rank Correlation Coefficient.

Given trials in which both targets were identified correctly, we found that in 19.59% (*SE* = .63) of the lag-1 trials, targets were reported in reversed order. For lag 3, this was 2.89% (*SE* = .32), and for lag 8, this was .45% (*SE* = .10). As tested with a GLMM, the number of order reversals in lag-1 trials where both T1 and T2 were reported correctly could be predicted by AB magnitude, *β* = .23, *SE* = .08, *z* = 2.73, *p* = .006. Thus, as shown in Fig 3, we found that a smaller AB magnitude was associated with fewer order reversals of T1 and T2.

### Pupil dilation

**Fig 4:**
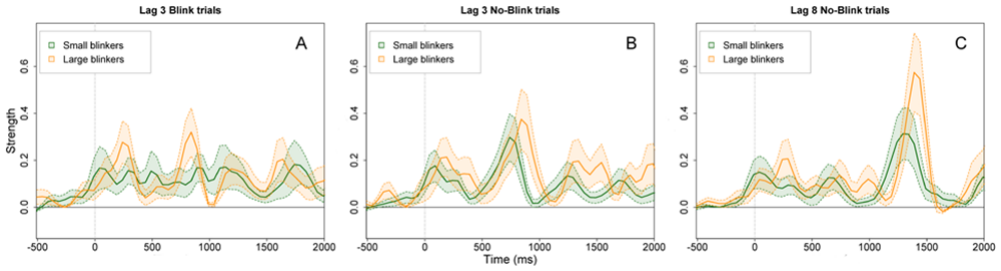
Deconvolved pupil dilation pulses. The mean strength of the deconvolved attentional pulses for small blinkers and large blinkers. For graphical purposes, the sample is divided in two groups based on the median AB magnitude. A) Lag-3 blink trials, i.e., T2 was identified incorrectly, B) Lag-3 no-blink trials, i.e., T2 was identified correctly, and C) Lag-8 no-blink trials. The depicted signal was smoothed with a Butterworth filter, and the x-axis was time-locked to the onset of T1. The error bars reflect the standard errors of the mean.

The deconvolved pupil data for lag 3 and 8 in either blink or no-blink trials are depicted in Fig 4A–4C. Here, for graphical purposes the sample was divided in two groups based on the median AB magnitude: small blinkers vs. large blinkers. However, the analyses were performed with AB magnitude as a continuous variable [38]. For the deconvolved pulses associated with attentional allocation to the targets, we tested to which extent latency and amplitude could be predicted by AB task performance. All analyses were performed under the prerequisite that T1 was reported correctly. Furthermore, in analyses regarding lag-8 trials or pulses associated with T2, we only analyzed no-blink trials.

#### T1 Latency

For lag-3 trials, in an LMM with AB magnitude and blink/no-blink trials as predictive factors, we only found a trend that AB magnitude was predictive for T1 latency, *β* = .14, *SE* = .07, *t* = 1.84, *p* = .068. Furthermore, there was no evidence that T1 latency differed between blink and no-blink trials (*p* = .18). However, when analyzing no-blink trials only, AB magnitude could predict T1 latency as tested with an LM, *β* = .25, *SE* = .10, *t* = 2.51, *p* = .015, but this was not the case when testing blink trials only (*p* = .76). Thus, individuals with a relatively small AB seem quicker to allocate attention to T1 in no-blink trials than individuals with a relatively large AB, but AB magnitude was not found to be related to T1 latency in blink trials.

In another LMM for lag-3 trials with mean T1 accuracy over all lags and blink/no-blink trials as fixed factors, mean T1 accuracy was predictive for T1 latency, *β* = -.24, *SE* = .08, *t* = -3.16, *p* = .002, such that higher mean T1 accuracy was associated with earlier allocation of attention to the first target. Again, there was no effect of blink/no-blink trials (*p* = .19). When analyzing no-blink trials and blink trials separately, an LM showed an effect of mean T1 accuracy over all lags in both no-blink trials, *β* = -.26, *SE* = .11, *t* = -2.39, *p* = .019; and in blink trials, *β* = -.23, *SE* = .11, *t* = -2.09, *p* = .040. Thus, mean T1 accuracy over all lags seems predictive for the timing of attention to T1 irrespective of T2 accuracy.

Regarding lag-8 trials, there was no evidence that T1 latency was related to AB magnitude, or mean T1 accuracy over all lags, as tested in two separate LMs (*p*s > .14). In addition, after visible inspection of the data (Fig 4C), we re-determined T1 latency in lag-8 trials using the local peak method instead of the first local peak method, as described in the method section. However, using the local peak method, there were also no significant effects of AB magnitude or mean T1 accuracy (*p*s > .17). Therefore, the relation between the timing of attention allocated to T1 and AB task performance seems apparent in lag-3 trials, i.e., during the blink period, but not at the longer lag.

#### T2 Latency

In lag-3 no-blink trials, as tested in two LMs, we found that both AB magnitude, *β* = .23, *SE* = .10, *t* = 2.25, *p* = .027; and mean T2|T1 accuracy at lag 3, *β* = -.25, *SE* = .10, *t* = -2.42, *p* = .018, were predictive for the timing of attention to T2. Furthermore, for lag-8 trials, T2 latency was also related to both AB magnitude, *β* = .25, *SE* = .09, *t* = 2.81, *p* = .006; and mean T2|T1 accuracy at lag 8, *β* = -.55, *SE* = .07, *t* = -7.70, *p* < .001. Thus, as can be seen in Fig 4B and 4C, individual AB task performance is reflected in the timing of attention to T2 in no-blink trials, such that better AB task performance predicts earlier allocation of attention to T2, irrespective of lag.

#### Delay

We tested whether the delay, defined as the time difference between the target onset and the local peak, differed between T2 and T1 in no-blink trials. For lag-3 trials, the delay was larger for T2 (*mean* = 441.27 ms, *SE* = 14.50) than for T1 (*mean* = 132.86 ms, *SE* = 15.00), *β* = 1.5, *SE* = .08, *t* = 18.16, *p* < .001; as was also the case for lag-8 trials (T1: *mean* = 162.08 ms, *SE* = 17.62; T2: *mean* = 493.09 ms, *SE* = 11.30), *β* = 1.61, *SE* = .10, *t* = 16.87, *p* < .001. These results indicate that the extra load of T2 processing on top of T1 processing causes a delay in the speed with which attention can be allocated to the targets.

#### T1 Amplitude

**Fig 5:**
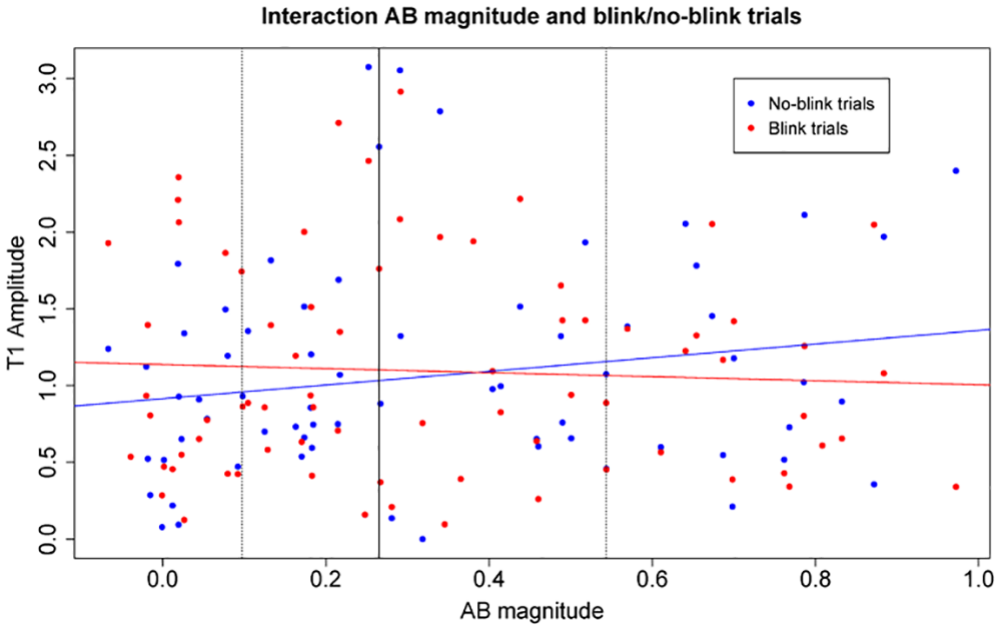
Interaction AB magnitude and blink/no-blink trials. Scatterplot depicting the interaction between AB magnitude and blink/no-blink trials for T1 amplitude in lag-3 trials. The vertical lines represent the quartiles of AB magnitude, where the solid vertical line represents the median AB magnitude.

For the analyses regarding T1 amplitude, we included in all models the two-way interaction term, because we set a-priori hypotheses about possible interactions between AB task performance and blink/no-blink trials. First, we analyzed the strength of T1 pulses at lag 3 with an LMM with AB magnitude, blink/no-blink trials, and their two-way interaction term as fixed factors. Note that one data point was removed from the analyses concerning T1 amplitude at lag 3, because the strength of this pulse associated with T1 processing deviated more than three standard deviations from the mean. Here, we found no unconditional main effect of AB magnitude or blink/no-blink trials (*p*s > .58), but there was a just significant interaction between AB magnitude and blink/no-blink trials, *β* = .21, *SE* = .10, *t* = 2.00, *p* = .049. This interaction is displayed in Fig 5, where it can be seen that small blinkers invest more attention in T1 in blink trials than in no-blink trials, but that for large blinkers this pattern is the other way around. However, a separate LM with AB magnitude as predictive factor for T1 amplitude in blink trials only did not reveal an effect of AB magnitude, *β* = -.05, *SE* = .11, *t* = -.45, *p* = .65. In addition, the same model for no-blink trials also showed no effect of AB magnitude, *β* = .16, *SE* = .11, *t* = 1.46, *p* = .15. Thus, the negative relation between AB magnitude and attentional investment in T1 in blink trials differs from the positive relation in no-blink trials, but the slopes per se were not significant. An LMM with mean T1 accuracy, blink/no-blink trials, and their interaction term did not reveal any significant effects (*p*s > .11).

#### T2 Amplitude

We found no evidence that T2 amplitude at lag 3 could be predicted by AB magnitude or by mean T2|T1 accuracy at lag 3 (*p*s > .72). In addition, there was no effect of AB magnitude or mean T2|T1 accuracy at lag 8 with regard to T2 amplitude at lag 8 (*p*s > .40).

## Discussion

The current study covered two goals that were both investigated by measuring pupil dilation. First, we aimed to reveal the time course of attentional allocation in the AB task in relation to individual AB magnitude. Behaviorally, we hypothesized that individuals with a smaller AB magnitude would maintain higher preservation of temporal order information [21]. Regarding pupil dilation, small blinkers were expected to allocate attention earlier in time to the targets, because they seem to be more efficient in target selection [21, 24], and earlier in WM updating [9, 16]. Second, based on the theory of attentional overinvestment to T1 as underlying the AB [4, 30, 31], we tested whether individual AB task performance is related to the amount of attention invested in selection of T1. We expected that attentional overinvestment to T1 would result in higher T1-elicited amplitudes in blink trials than in no-blink trials. Furthermore, if attentional overinvestment would be related to individual AB task performance, individuals with a relatively large AB magnitude should also show a larger attentional response to T1 compared to small blinkers.

In a sample of 90 participants, we found large differences in individual AB task performance of which the distribution is graphed in the boxplots in Fig 2A–2D. Furthermore, we found that a smaller AB magnitude was related to fewer reversed order reports of the targets in lag-1 no-blink trials. In the pupil data, we found for lag 3 that timing of attention to T1 could be predicted by both AB magnitude and mean T1 accuracy in no-blink trials, but only by mean T1 accuracy in blink-trials. For lag 8, attentional timing to T1 was not related to AB task performance. Timing of attention to T2 could be predicted by AB task performance irrespective of lag. Regarding attentional overinvestment in T1, we found an interaction between blink/no-blink trials and AB magnitude. That is, a larger AB magnitude was related to higher attentional investment in no-blink trials than in blink trials, whereas this pattern was the other way around for small blinkers. When analyzing blink and no-blink trials in separate models, there was in both cases no effect of AB magnitude.

### Timing of attention

Behaviorally, we revealed that a smaller AB magnitude is related to higher preservation of temporal order information regarding T1 and T2. This replicated an earlier study [21], where we also showed a positive relation between AB magnitude and the number of reversed order reports. These results refute the theory of [39] that the AB can be seen as a cognitive strategy that enforces episodic distinctiveness between successive stimuli. According to this theory, a smaller AB would result in more order reversals, because episodic distinctiveness would be lower for these individuals. However, we observed the exact opposite pattern in the current study and in [21]. As suggested by Akyürek et al. [40], order reversals at lag 1 might reflect a mechanism of temporal integration, where targets are integrated into one temporal event when they are presented in close temporal succession. Given that large blinkers make more order reversals, they may also be more prone to integrate information than small blinkers. Future research is needed to investigate this idea in closer detail.

By measuring pupil dilation, we showed that when both targets are identified correctly, timing of attention to T2 could be predicted by either AB magnitude or target accuracy at the tested lag. That is, individuals with a relatively small AB magnitude were quicker to allocate attention to T2 than individuals with a large AB magnitude. This finding is in line with earlier pupil dilation findings where attentional timing to T2 was also related to mean T2|T1 accuracy in no-blink trials [29]. Furthermore, in EEG studies, the P3 component was found to peak earlier for non-blinkers than for blinkers in no-blink trials regardless of target and lag [9, 16]. Therefore, it seems that earlier allocation of attention in response to T2 is beneficial for general AB task performance. However, whether this earlier timing of attention is due to faster allocation of attention, better preparation of the attentional system, or both remains to be clarified in further research.

For T1, the relation between attentional timing and AB task performance seems to be somewhat less consistent. For the short lag, both mean T1 accuracy over all lags and AB magnitude could predict attentional timing to T1 in no-blink trials, but only mean T1 accuracy was related to attentional timing to T1 in blink trials. These findings suggest that identification of T1 is dependent on the timing of attention to the first target irrespective of T2 identification. However, in no-blink trials, earlier timing of attention to T1 is also predictive for a smaller individual AB magnitude. If the second target is not identified correctly, or presented at lag 8, the timing of T1-allocated attention could no longer be linked to AB magnitude. It must be noted that this latter finding may be due to higher variability of attentional timing to T1 in blink lag-3 trials and no-blink lag-8 trials. That is, because in these trials T2 is either missed or presented at the long lag, timing to T1 is less crucial, i.e., resulting in a mixture of trials on which participants are either early or late in allocating attention to T1. In no-blink lag-3 trials, late allocation will have more consequences for T2 performance and will often result in missing T2, thus, resulting in less variability in attentional timing. This increased variability in blink lag-3 trials and no-blink lag-8 trials may explain why tests remained non-significant despite visual inspection of Fig 4A and 4C suggesting otherwise.

In an earlier pupil dilation study, we did not find any evidence for a relation between the timing of attention to T1 and AB task performance [29]. It must be noted, though, that these analyses comprised both blink and no-blink trials, which in this study also resulted in only a trend for AB magnitude. Given that EEG studies did find an earlier peak of the T1-elicited P3 for non-blinkers than for blinkers [9, 16] in no-blink trials, the relation between attentional timing to T1 and individual AB task performance remains subject for further research. However, the current evidence suggests that earlier attentional allocation to T1 can at least be predicted by AB magnitude in short-lag no-blink trials.

Finally, we found a difference between T1 and T2 in the delay of attentional timing to the targets defined as the difference between target onset and the peak associated with attentional allocation to the target. The delay of attentional timing to the targets was found to be larger for T2 than for T1 at both lag 3 and 8 no-blink trials, which suggests that the additional processing of T2 on top of T1 processing increases the workload, and causes a delay in the timing of attention allocated to T2. This is in line with previous studies reporting WM consolidation to be delayed for T2 compared to T1 [9, 41].

### Attentional overinvestment

We found that the pattern for attentional investment in T1 differs in relation to individual AB magnitude, such that small blinkers invested more attention in blink trials than in no-blink trials, and that large blinkers invested more attention in no-blink trials than in blink trials. However, AB magnitude was not found to be predictive for T1 amplitude in either blink trials or no-blink trials when tested separately. Several earlier studies have revealed evidence for the existence of attentional overinvestment as an aspect underlying the AB [4, 30, 31]. However, based on these results, large blinkers in particular would be expected to invest more attention in T1 in blink trials than in no-blink trials. However, we found this expected pattern only for the relatively small blinkers, and the exact opposite pattern for large blinkers. It remains unclear why these specific patterns were found. In earlier studies measuring pupil dilation, and using the pupil dilation deconvolution method, we did find significant differences in amplitude for the T1 pulse between blink trials and no-blink trials [27, 29], suggesting that the technique itself is sensitive enough to detect differences in attentional investment. Note also that we tested a large sample of participants with a classic AB paradigm which resulted in the typical hook-shaped AB pattern, as can be seen in Fig 1.

Finally, it should be noted that a potential limitation of the individual differences approach is that the proportion of blink/no-blink trials is different for participants who performed well in the AB task versus participants who performed less optimal. To alleviate the influence of this possible confound, we tested both a large sample of 80 participants and a reasonable number of 56 trials per lag. Furthermore, we used a robust analyzing technique, i.e., linear mixed models, that can handle potential differences in variance.

### Conclusion

Based on the current findings, we conclude that there is a negative relationship between AB magnitude and preservation of temporal order information, such that small blinkers seem better able to preserve temporal order information. Furthermore, in trials during which both targets are successfully identified, faster attentional allocation to T2 is predictive for better AB task performance. The relation between attentional timing to T1 and individual AB task performance was less consistent across conditions, but earlier attentional allocation to T1 could at least be predicted by AB magnitude in short-lag no-blink trials. Finally, we did not find evidence for the idea that large blinkers tend to invest more attention to T1 than small blinkers. Therefore, it seems that timing of attention rather than the amount of allocated attention to targets is the most important factor to account for individual differences in the AB.

## Supporting Information

S1 Data The behavioral data. (TXT)

S2 Data Deconvolved pupil dilation data including the peaks and their amplitudes as used to analyze the pupil response. This dataset regards the peaks and amplitudes associated with attentional allocation to T1.
(TXT)

S3 Data Deconvolved pupil dilation data including the peaks and their amplitudes as used to analyze the pupil response. This dataset regards the peaks and amplitudes associated with attentional allocation to T2 at lag 3.
(TXT)

S4 Data Deconvolved pupil dilation data including the peaks and their amplitudes as used to analyze the pupil response. This dataset regards the peaks and amplitudes associated with attentional allocation to T2 at lag 8.
(TXT)
